# Metal-Organic Framework (MOF)—A Universal Material for Biomedicine

**DOI:** 10.3390/ijms24097819

**Published:** 2023-04-25

**Authors:** Andrey A. Vodyashkin, Antonina V. Sergorodceva, Parfait Kezimana, Yaroslav M. Stanishevskiy

**Affiliations:** 1 Institute of Biochemical Technology and Nanotechnology, Peoples’ Friendship University of Russia (RUDN University), 6 Miklukho-Maklaya Str., 117198 Moscow, Russia; sergorodceva.1997@mail.ru (A.V.S.); kezipar@outlook.com (P.K.); stanishevskiy-yam@rudn.ru (Y.M.S.); 2 Department of Agrobiotechnology, Peoples’ Friendship University of Russia (RUDN University), 6 Miklukho-Maklaya Str., 117198 Moscow, Russia

**Keywords:** metal-organic frameworks, synthesis and post-synthetic modification, biomedical applications, targeted delivery, diagnostic systems

## Abstract

Metal-organic frameworks (MOFs) are a very promising platform for applications in various industries. In recent years, a variety of methods have been developed for the preparation and modification of MOFs, providing a wide range of materials for different applications in life science. Despite the wide range of different MOFs in terms of properties/sizes/chemical nature, they have not found wide application in biomedical practices at present. In this review, we look at the main methods for the preparation of MOFs that can ensure biomedical applications. In addition, we also review the available options for tuning the key parameters, such as size, morphology, and porosity, which are crucial for the use of MOFs in biomedical systems. This review also analyses possible applications for MOFs of different natures. Their high porosity allows the use of MOFs as universal carriers for different therapeutic molecules in the human body. The wide range of chemical species involved in the synthesis of MOFs makes it possible to enhance targeting and prolongation, as well as to create delivery systems that are sensitive to various factors. In addition, we also highlight how injectable, oral, and even ocular delivery systems based on MOFs can be used. The possibility of using MOFs as therapeutic agents and sensitizers in photodynamic, photothermal, and sonodynamic therapy was also reviewed. MOFs have demonstrated high selectivity in various diagnostic systems, making them promising for future applications. The present review aims to systematize the main ways of modifying MOFs, as well as the biomedical applications of various systems based on MOFs.

## 1. Introduction

The use of different nanomaterials in biomedicine has become increasingly relevant in recent years, as they are used in different aspects, including diagnostics, therapy, and prevention of various diseases [1,2,3]. For a long time, polymers were used as carriers of drugs, in diagnostics, and other applications. It is also worth noting the use of various polymeric materials as coating agents, which are actively used in wound care and after surgery. Hybrid materials are also created based on polymeric materials, allowing the combination of the positive qualities of both materials [4,5,6].

Nanoparticles are another material that has been actively used for biomedical purposes in recent years. Nanoparticles are a relatively new material in the medical field, but over the years they have become more and more relevant, since they have a very wide range of applications due to their different nature. Metal nanoparticles can be actively used as antibacterial, cytotoxic, and diagnostic materials [7,8,9,10]. Polymer nanoparticles can also be used as drug delivery systems to diagnose and treat various diseases [4,11].

More recently, metal-organic frameworks (MOFs), a class of hybrid porous materials composed of organic metal ions bonded with organic inclusions, have become promising for applications in nanotechnology and bionanotechnology due to their physical and chemical properties. They have high sorption capacity, an actively developed porous surface, thermal stability, chemical stability, modifiability, and are easily functionalized with pH, and ion sensitivity. The combination of these properties allows for promising applications of MOFs in a wide range of industries and technologies. Over the past two decades, metal-organic framework structures have found application as multifunctional catalysts for various purposes. They have been used for heterogeneous organic reactions [12,13,14,15], photocatalysis [16], gas storage and separation [17,18,19], energy storage and conversion [20,21,22], and intracellular molecular sensing for biomedical purposes [23,24]. MOFs also provide a promising new platform for creating polar crystalline materials that have technologically important physical properties relevant to fields ranging from optics to electronics [25]. In addition to their use as porous materials, MOFs are also potential candidates for applications in thin-film materials [26].

In recent years, the biomedical applications of various metal-organic systems have become more relevant [27,28,29]. The application of MOFs for biomedical purposes is often associated with the complexity of obtaining them in a given shape and size that is comparable with biological objects (up to 200 nm), as well as ensuring biocompatibility, biodegradability, and great functional and target relevance [30,31].

The stability of MOFs in physiological fluids is also important for biomedical applications. For example, some reports have shown that some zinc-based MOFs are unstable and rapidly lose their structural integrity and large surface area when immersed in water [32]. Additionally, the use of some MOFs may raise concerns due to the potential formation of toxic metal ions (Cr^3+^, Cd^2+^, etc.) and other harmful components during MOF biodegradation [33].

Various applications of MOFs for biomedical purposes have been presented by researchers. Due to the high functionality of the large set of ligands that can be used in the creation of MOFs, they are one of the most promising materials that can soon be used in various fields, including biomedical applications [34]. It is worth noting that the biomedical field requires specific conditions and stricter requirements for the materials used. Therefore, the systematization and actualization of modern methods of synthesis, modification, and tuning of the key parameters of MOFs are relevant. It is also especially important to present the main applications of MOFs in various fields of health care and medicine.

**Figure 1:**
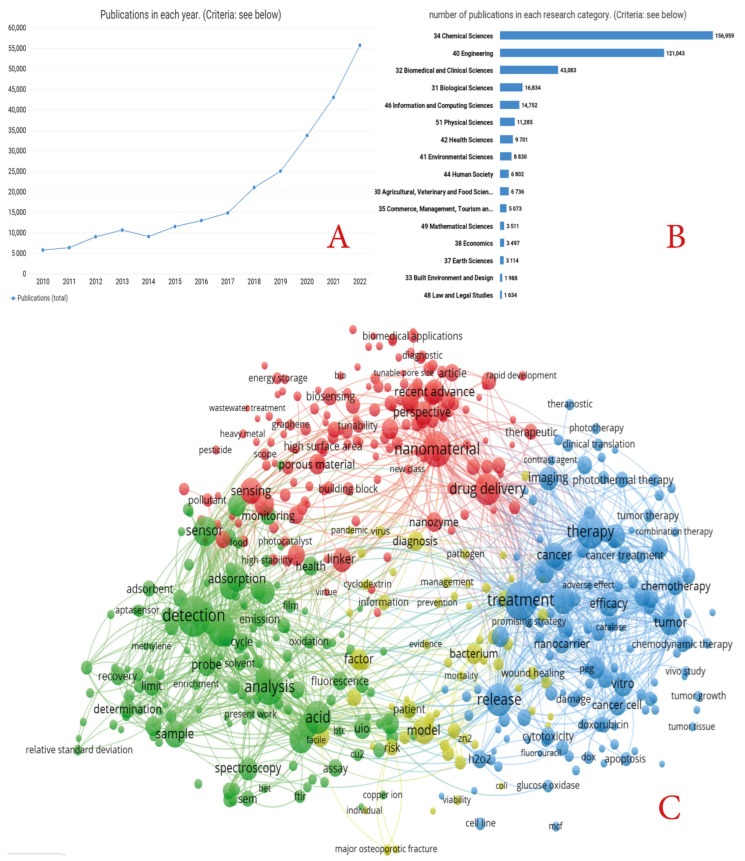
(**A**) Number of publications on the request of MOFs in 2012–2022 (according to dimensions). (**B**) Main research directions within the scope of MOFs (according to dimensions in 2012–2022). (**C**) Histogram of the main terms used in the titles and abstracts of articles devoted to biomedical applications of MOFs (based on dimensions in 2012–2022).

Moreover, the great potential of MOFs has been demonstrated by the sharp increase in publications related to MOF research in the last seven years (Figure 1A, dimensions data). In recent years, more new production methods have been developed, which in turn have led to the development of new applications. Figure 1B shows that biological and medical applications are among the leading topics of application. However, from the many reviews published on MOFs, most focus on specific, narrow applications of MOFs, with very few reviews dedicated to the complex biomedical applications of MOFs. Therefore, we present a complex analysis of the main methods for preparing MOFs, including possible modifications and changes of MOF parameters, and the main applications of MOFs in the biomedical sphere, as highlighted in Figure 1C. This figure is based on the frequency of the mention of terms in the name and abstracts (dimensions data).

For this review, electronic scientific databases such as PubMed, Science Direct, Web of Science, Scopus, and Medline were used to analyze studies on MOFs, their synthesis, post-synthetic modifications, and biomedical applications. Data were sorted from 2012–2022 to provide the latest and most current information. However, when there was a need for more clarification, other data were also used.

## 2. General Approaches and Methods of Synthesizing MOFs for Biomedical Purposes

The synthesis of metal-organic compounds directly affects the crystallization of the MOFs’ structure and determines its properties and functional characteristics [35]. Various synthetic techniques allow the framework topology, pore structures, and size of the MOFs to be altered by selecting specific metal centers and organic linkers. The chemical properties of the resulting materials can be transformed by the chemical functionalization of linkers and post-modifications [36].

The synthesis methods and conditions affect the functional and structural properties of the resulting materials. Various physical and chemical synthesis approaches are used to create metal-organic framework structures, taking into account parameters such as temperature, reaction time, pressure, pH, solvent, etc. [37,38,39].

For biomedical purposes, MOFs of multivalent metals such as zirconium (IV) [40], iron (III) [41,42], zinc (II) [43], and copper (II) [44] are more applicable. The ligands used in MOF synthesis usually have several carboxylic or amine functional groups that depart either from the alkyl chain or from a ring structure such as benzene or imidazole [45]. Coordination of the ligand with the ion results in a crystal lattice with a regular repeating geometry [46].

### 2.1. Solvothermal and Hydrothermal Methods

The most common method for obtaining metal-organic compounds is solvothermal/hydrothermal synthesis, which provides a variety of morphologies for the obtained structures [47]. The experimental technique involves the interaction of a metal salt and an organic linker, which are dissolved in a solvent and placed in a closed reaction vessel for the formation and self-assembly of MOF crystals. Usually, solvents such as N,N-dimethylformamide, N,N-diethylformamide, methanol, ethanol, acetone, and acetonitrile are used for the synthesis. The synthesis temperature is usually below 220 °C, and the crystallization time varies from several hours to several tens of days [48].

The effect of these four parameters during solvothermal synthesis has been extensively investigated on MOF-5. The authors showed that the formation of the pure MOF-5 phase proceeded mainly as a two-step process at temperatures above 130 °C. Moreover, metastable intermediate solid phases obtained initially from the solution phase during metal stirring had a marked effect on MOF-5 formation (e.g., bulk MOF-5 phases). Because the m/L ratio exceeded 1.33, MOF-5 formation was prevented by the presence of excess terephthalic acid. The addition of water during the synthesis of MOF-5 resulted in the formation of MOF-69c, whereas MOF-5 could still be formed after the addition of terephthalic acid [49].

**Figure 2:**
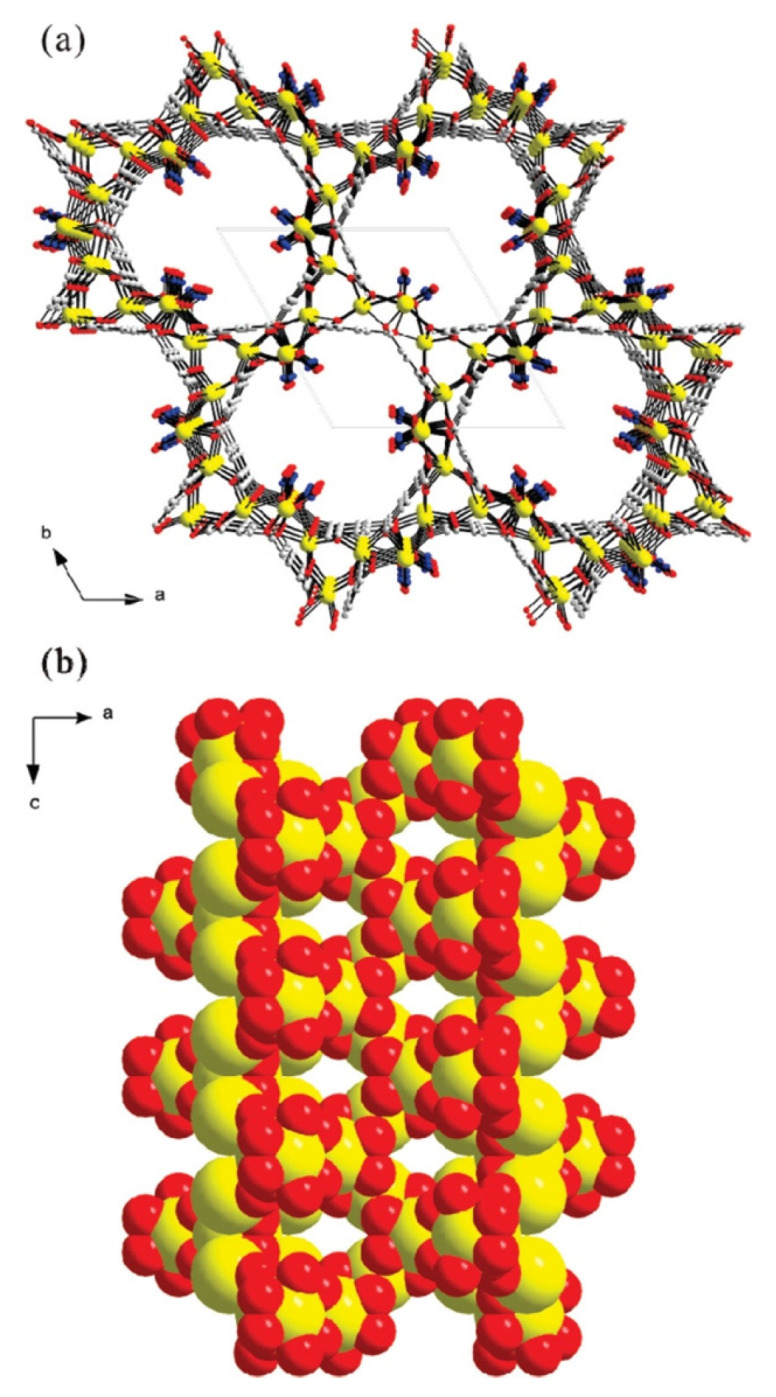
Models of non-centrosymmetric strontium–organic framework material representing helical nanotubular channels in (**a**) the ab-plane and (**b**) the ac-plane, respectively (yellow, Sr; gray, C; blue, N; red, O), reprinted from [50].

Lee et al. synthesized a non-centrosymmetric strontium-organic framework material under solvothermal conditions, which has a helical channel structure consisting of strontium oxide polyhedral and a 1,3,5-benzoltricarboxylate linker (Figure 2) [50]. The solvothermal reaction was carried out by combining N(CH_3_)_4_Cl, Sr (NO_3_)_2_, 1,3,5-benzoltricarboxylic acid, and HNO_3_, HCON(CH_3_)_2_, and then autoclaving the mixture at 180 °C for 3 days. The resulting structure had high thermal stability up to 520 °C and broke down to SrCO_3_. The high thermal stability was found to be related to the flexibility of the liquor molecules, coordination-related interpenetration, and the highly symmetric structural environment of the MOF.

### 2.2. Microwave Synthesis

Currently, microwave synthesis is increasingly being used to obtain various materials [51,52]. The use of microwave radiation is also used to prepare various MOFs [53]. Exposing the reaction mixture to microwaves causes a combination of thermal effects induced by a high heating rate, local overheating or “hot spots”, and selective absorption of microwave radiation by various molecules. The combination of these effects provides a unique conditions that can reduce crystallization time and increase the ability to control phase, morphology, and particle size distribution.

The principle underlying synthesis with microwave radiation is based mainly on the interaction of electromagnetic radiation with electric charges, which may include polar ions and solvent molecules or electrons. In the liquid phase, as the temperature increases, the kinetic energy of the molecules increases, which leads to increased collisions between polar molecules when frequency is used in the electromagnetic field [54,55].

**Figure 3:**
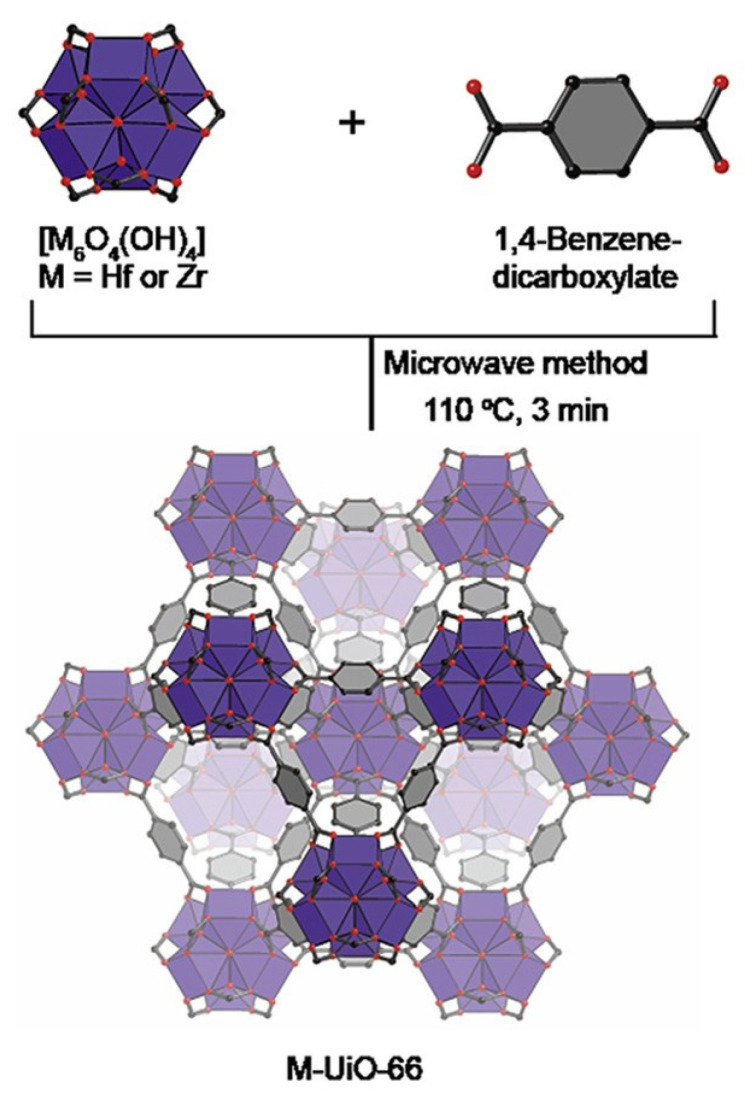
Microwave method for effective and fast synthesis of nano metal-organic frameworks, reprinted from [57].

Wu et al. analyzed MOF-74 obtained by solvothermal and microwave methods. They showed that the microwave method produces a larger specific surface area and micropore volume with a similar average pore diameter for MOF-74. In addition, the MOF-74 produced by the microwave method had a higher gas separation capacity than the MOF produced by the solvothermal method [56]. Other studies confirmed that the use of microwave radiation in the synthesis process can produce metal-organic framework structures with a relatively larger surface area and specific pore volume than those obtained by conventional methods. Moreover, the duration required to obtain MOFs was only 3 min (Figure 3) [57].

A variety of MOF structures have been obtained using microwave radiation [58]. Taddei et al. also confirm that MOFs obtained by thermal and microwave methods do not differ in properties, morphology, and structure [59]. In their work, Jhung et al. demonstrate the feasibility of using microwave radiation as a phase-selective and very fast method for MOF synthesis [54].

It is worth noting that, despite several advantages, microwave methods are not currently widespread methods for obtaining metal-organic framework.

### 2.3. Ultrasonic Method

One particularly interesting method for the synthesis of metal-organic framework structures is the sonochemical method, which allows for significantly accelerated reactions, improved environmental friendliness, and energy efficiency of the processes. It is an easy-to-use method that can be applied at room temperature.

Sonochemistry deals with changes in the physical and chemical properties of molecules subjected to powerful ultrasonic irradiation (20 kHz–10 MHz) [60]. Ultrasound induces chemical or physical changes during cavitation, a phenomenon involving the formation, growth, and instantaneous explosive collapse of bubbles in a liquid. This can create local hot spots with temperatures of approximately 5000 °C, pressures of 500 atm, and reaction times of several microseconds [61]. Such extreme conditions stimulate the chemical reaction process and promote the formation of nanoscale particles, mainly due to the instantaneous formation of multiple crystallization nuclei.

Using the ultrasonic method, metal-organic compounds such as MOF-5 [62], Fe-MIL-53 [63], and MOF-177 [64] can be synthesized with significantly reduced synthesis time (about 30 min) compared to conventional solvothermal synthesis (24 h). The sonochemical method of synthesis also leads to homogeneous nucleation of crystallization in the resulting compound, which is an important advantage compared to other synthesis methods.

### 2.4. Mechanochemical Synthesis of MOFs

The mechanochemical method (milling) of synthesis is carried out using metal precursors and bridging organic ligands, whose interaction leads to a chemical reaction resulting in the formation of coordination complexes with a reorientation of intramolecular bonds [55]. The chemical transformation and formation of the metal-organic complex are preceded by a mechanical breakdown of intramolecular bonds. There are three different mechanochemical methods for creating MOFs: pure milling, liquid-assisted milling, and ion-liquid-assisted milling [65]. Mechanochemical synthesis is more environmentally friendly due to the lack of solvents. Moreover, when using metal oxides, only water is produced as a byproduct [66]. In addition, it is worth noting the relatively faster synthesis time of the obtained materials and the lack of use of elevated temperatures also increases the environmental friendliness of the processes.

Al-Terkawi et al. synthesized dicarboxylate metal-organic compounds based on alkali earth metals using the mechanochemical method. The mechanochemical synthesis was carried out by grinding the precursors Sr(OH)_2_-8H_2_O with tetrafluorophthalic acid or phthalic acid in a molar ratio of 1:1 [67]. In addition, it has been shown that the water content has a key influence on the grinding time and condition of the resulting MOFs: the lower the water content, the longer the grinding time required [68].

### 2.5. Other Synthesis Methods

Other techniques used for the synthesis of metal-organic compounds, such as the electrochemical method [69], atomic layer deposition [70], ionothermal method [71,72], sol-gel method [73,74,75,76], and the expanded supercritical fluid solutions method [77] are rarely used and are currently being studied.

The main advantage of the electrochemical synthesis method is the absence of counter ions such as nitrate, perchlorate, or chloride from metal salts; therefore, the obtained materials will have higher purity [78]. The synthesis process uses short reaction times and milder conditions [79], which makes this method more affordable for industrial large-scale production of metal-organic framework compounds [80]. In their work, Joaristi et al. obtained the most common metal-organic compounds, namely HKUST-1, ZIF-8, MIL-100(Al), MIL-53(Al), and NH2-MIL-53(Al) by anodic dissolution in an electrochemical cell using electrochemical synthesis [79]. They also investigated the effect of various reaction parameters such as solvent, nature of the electrolyte, current voltage, and temperature on the yield and physicochemical properties of the obtained compounds. They demonstrate that electrochemical synthesis is a reliable method whose main advantages are shorter synthesis time, milder conditions, and easy synthesis of nanoscale MOFs.

The sol-gel synthesis method is a versatile strategy for the preparation of functional inorganic and hybrid materials that facilitates control of the molecular composition relevant to basic and applied research [73]. Recent advances have made it possible to use this method for the synthesis of functional porous materials. The metal-organic framework MOF-5 was synthesized by the sol-gel method using SiO2 nanoparticles with a given surface morphology as nucleation agents [75]. Modified silicon dioxide nanoparticles are excellent nucleation agents that contribute to the formation of microcrystals in a monodispersed metal-organic framework. The formation rate of MOFs stimulated by nanoparticles as sol-gel can be ten times higher than the solvothermal method of synthesis.

A monolithic organometallic framework was also synthesized using the sol-gel method, which can be used for HKUST-1 methane sorption without the use of binders and high pressures [76]. The mild conditions of the synthesis process led to a dense monolithic structure of the resulting material. In sol-gel synthesis, HKUST-1 was able to retain its characteristic powder structure and porosity with a uniform particle size of 51 nm while showing three times the density and a higher volumetric gas adsorption capacity.

In the sol-gel preparation of metal-organic compounds, it is important to better understand how sol-gel processing parameters, including precursor, catalyst, and solvent identity, as well as reaction conditions (temperature, concentration, and reaction time) affect the composition, structure, and properties of the resulting materials. Currently, there is no systematic information that structures this knowledge and gives a clear impetus to the use of colloidal systems for producing MOFs.

Another method based on the colloidal properties of the solution is the ionothermic method. Ionothermal synthesis is based on the use of an ionic liquid (IL) as a solvent and base for the preparation of a variety of crystalline solids, including MOFs [81]. Compared with traditional methods of synthesizing organometallic compounds, the transition from the molecular reaction medium to the ionic one leads to the appearance of new types of materials with different structural properties [71]. This can be directly related to the chemistry of the IL, which is the main advantage of this method of MOFs synthesis. It is worth noting that this method is very rarely used to obtain various MOF structures.

The supercritical fluid synthesis method offers several specific physical and chemical advantages as an alternative solvent for the production of functional porous materials [82,83]. The absence of surface tension and capillary stresses in the supercritical fluid state facilitates the formation of nanostructured metal-organic materials without destroying the brittle pore network [77]. Drying and impregnation techniques using supercritical CO_2_(scCO_2_) are an effective approach for synthesizing and improving nanostructured porous materials [84].

### 2.6. Post-Synthetic Modification of MOFs for Biomedical Application

Post-synthetic modification is a process designed to modify MOFs or produce new materials (with new properties and parameters) that retain the characteristic features of MOFs, including high crystallinity, a large surface area, and a structure consisting of very regular metal-ligand bonds. By modifying MOFs post-synthetically, additional properties can be introduced that otherwise cannot be obtained through synthesis, making them more applicable to the biomedical field [85,86,87]. One of the advantages of performing chemical modification is the ability to incorporate the necessary functional groups into the obtained material rather than during the synthesis process (the monomer molecules are not involved in the reaction) [88]. Thus, the newly introduced functional groups (and the reaction conditions required for the introduction of these groups) should only be compatible with the final material, and there is no need to clarify the effect of these molecules (groups) on the MOF synthesis process [89].

MOFs contain an organic ligand component (unlike many other crystalline, inorganic solids), and thus open the door to a wide range of organic transformations [90]. Because MOFs are very porous, the ability of reagents to penetrate solids suggests that functionalization can be achieved both inside and outside of the resulting material [91]. In addition, the ability to treat the surface with various polymers (PEG, starch) can increase the hydrophilicity of MOFs and thereby increase their affinity for biological systems [92].

Methods for performing post-synthetic modification (PSM) of MOFs can be divided into three areas: (a) metal-based PSM, (b) ligand-based PSM, and (c) post-synthetic guest substitution in MOFs [93]. The type of chemical bond that is formed or broken during the post-synthetic approach distinguishes each of these methods. It is important to note that these different post-synthetic methods are not mutually exclusive; on the contrary, the use of several combined methods of post-synthetic approaches gives better results in obtaining material with certain functional properties [94].

**Figure 4:**
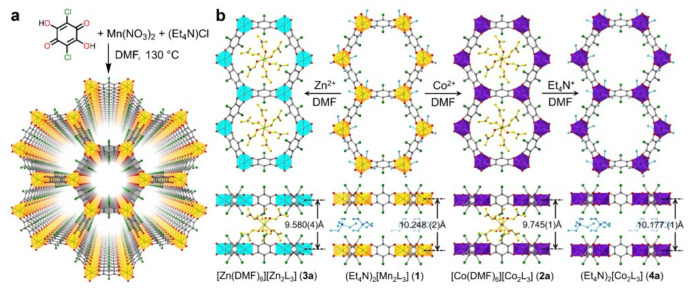
(**a**) Synthesis and structure of the manganese-benzoquinoid framework. (**b**) Scheme depicting the single crystal-to-single-crystal conversions via metal and counterion exchange, reprinted from [95].

Post-synthetic metal substitution is a unique means of creating new MOFs that cannot be obtained through classical synthetic methods due to low reaction rates or difficulties in forming a crystal structure with the ligand. Liu et al. demonstrated the possibility of post-synthetic modification of the manganese-benzoquinoid framework by replacing the metal with cobalt and zinc ions (Figure 4). In their work, they studied in detail the mechanism, kinetics, and extent of metal substitution during PSM, as well as the MOF obtained after modification [95]. It is worth noting that the possibility of metal replacement with Co^2+^ and Zn^2+^ (both partial and complete) could have a positive impact on the biomedical applications of MOFs and could also create unique bimetallic systems that could have the advantages of both metals.

**Figure 5:**
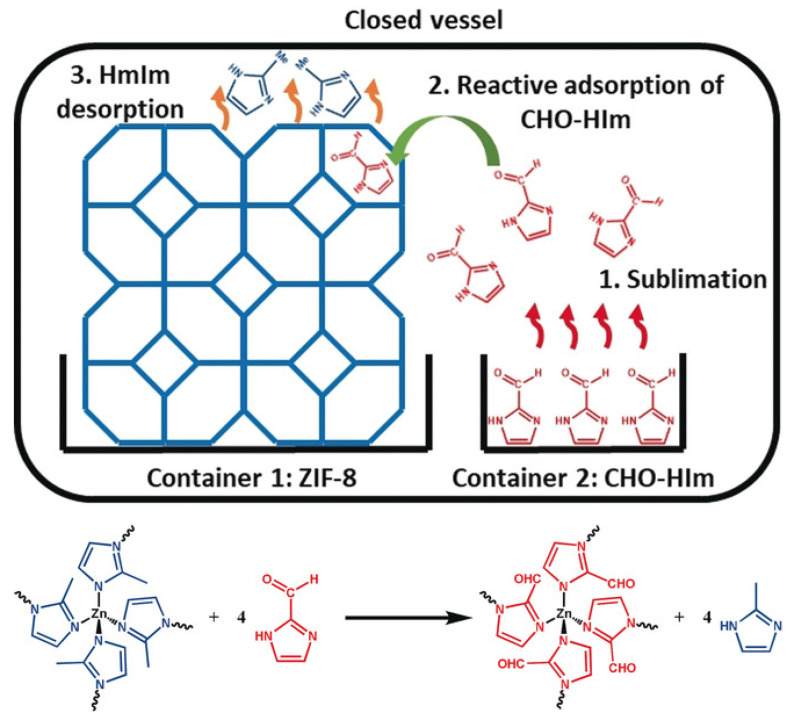
Schematic representation of the vapor–phase linker exchange process, reprinted from [96].

Ligands are the basis of the framework structure of MOFs and largely determine the structure and parameters of the resulting materials. New ligands can form frameworks similar to the original structure or can give structures with different dimensions. Replacing the ligand also makes it possible to functionalize the MOF and give it new properties. Marreiros et al. demonstrated the possibility of replacing the MOF ligand ZIF-8 in the vapor phase (Figure 5) [96]. This process consisted of three stages: (1) adsorption of the incoming linker, (2) exchange of protons between the adsorbed incoming linker and the linkers in the ZIF-8 framework, and (3) removal of the protonated methyl imidazole ligand. The authors studied the kinetics and critical parameters of this method, which forms an important basis for the PSM of various imidazole MOFs. Through these processes, the use of MOFs will help to form a set of processing tools for the microfabrication of chemical sensors used for biosystems, among other applications [96].

Post-synthetic modification can be used to remove solvent or synthesis products that may be present in the pores of the MOF after synthesis [97]. This process helps to increase the surface area of the MOF and improve its safety, which are defining indicators in the application of MOFs for biomedical purposes. In addition, this process can increase the electrical conductivity and magnetic properties of MOFs, which have a key impact on the possibility of creating in vivo sensors for the diagnosis (theranostics) of various diseases [98].

It is worth noting that methods of simultaneous post-synthetic modification have now been developed, for example, to replace both metal and ligand [99,100]. The creation of materials with desired properties, as well as the improvement of existing ones, confirms the importance of post-synthetic modification after MOF synthesis. Despite their usefulness, post-synthetic methods require careful consideration of the reaction conditions as well as product characterization to ensure that the reaction proceeds to yield the target product.

### 2.7. Adjustment of Main Factors for Biomedical Applications of the MOFs

Ahmadi et al. [101] have shown that the size, porosity, and morphology of MOFs are key physical parameters that determine their potential for biomedical applications. Currently, there are methods available to successfully control, modify, and tune these parameters. Parameter modification can be performed both during synthesis and during the post-synthetic modification steps.

#### 2.7.1. Size

Size is a key characteristic in determining the properties and applications of MOFs in biomedical fields. Suitable additives (modulators) can control the size of MOF particles in the reaction. In addition, by modulating nucleation and growth processes, synthesis methods and experimental parameters effectively influence crystal size. MOF particle size can be controlled by optimizing the synthesis parameters, such as adjusting the initial concentration of precursors and adding co-modulators or capping ligands to the synthesis medium [102]. The particle size of Zr-based MOFs is regulated by the addition of dodecanoic acid (DA) and triethylamine (TEA) as co-modulators. In UIO-66 MOF synthesis, monocarboxylic acids and organic bases can serve as modulators to regulate particle size. In their work, Wang et al. demonstrated that TEA can be used as a nucleation modulator to control particle size. It has been reported that TEA can reduce the nucleation time of crystallization by accelerating the deprotonation of the BDC (benzoldicarboxylate) ligand to produce smaller particles [103].

In their work, Nguyen Thi et al. added different amounts of polyethylene glycol 400 (PEG) directly to the reaction mixture to control the size of the MIL-53(Fe) MOF produced by ultrasound. They found a correlation between the concentration of PEG added to the mixture and the size of the MOF [104]. This work confirms that different modulators can be successfully used for size control in various methods of MOF production. It is worth noting that the use of PEG in this system not only reduces the size of the MOF, but also increases the specific surface area and thus maximizes the loading of the MOF with drug substances.

In addition to using additional modulators to control the size of the MOFs, the METAL-LIGAND ratio can also be used. As the ratio increases toward the ligand, the size of the MOF decreases [105]. It was shown that the ligand covers the surface, thereby dramatically reducing the concentration of metal ions and stopping local crystal growth [106]. It is worth noting that the strategy of using excess ligands cannot be applied to all MOFs and must be tailored to each individual system [107]. The effect of ligand excess on the size of some imidazole ZIF MOFs has been proven [108]. Reducing the concentration of reagents in the reaction mixture can also be used to reduce the size of MOFs, which can be achieved by decreasing the concentrations of both reagents in the system, thus leading to the formation of fewer bonds, and allowing the system to isolate itself from additional metal ions [105].

#### 2.7.2. Porosity; Morphology

**Figure 6:**
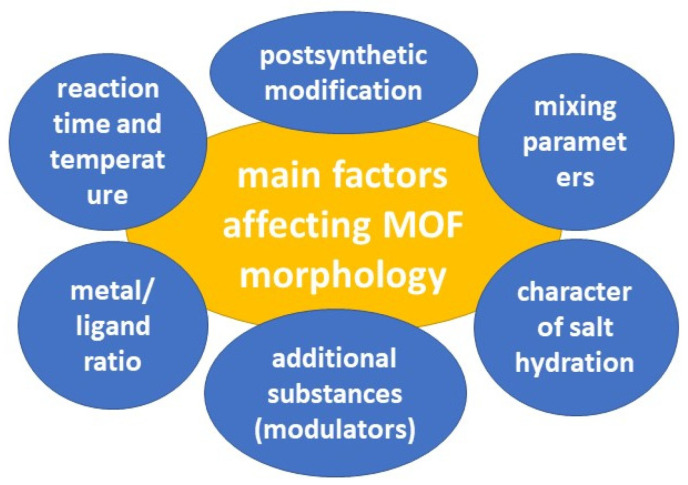
The main factors affecting MOF morphology.

The pores of MOFs can be rigid or more flexible, depending on the MOF composition and guest-host interactions [109]. MOF pores exhibit respiration, swelling, ligand rotation, and subnetwork shifts. MOF respiration, in which the unit cell structures change upon binding to loaded molecules, has been linked to the loading and release of molecules such as drug substances [110]. Swelling, in which the unit cell expands while retaining its shape, also depends on the guest-host interaction and is associated with drug release. The rotation of the ligand occurs around the metal coordination centers, which allows the pore parameters to change [111]. Subnetwork displacements can occur when binding forces are relatively weak, allowing the MOF components to move, drift, and shift [46]. Several factors can influence the morphology of the resulting MOFs (Figure 6).

**Figure 7:**
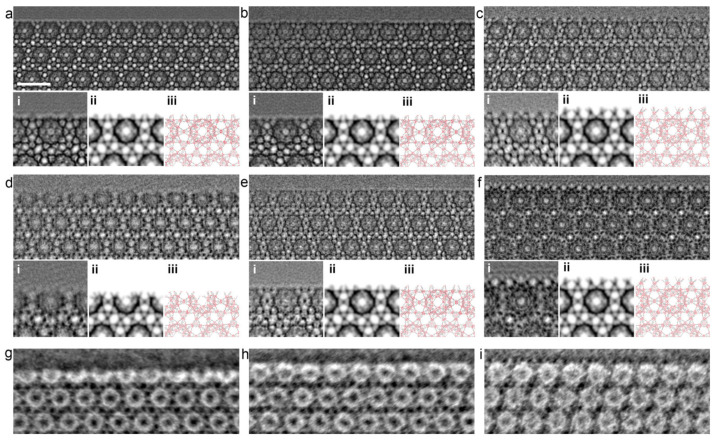
HRTEM images of freshly synthesized samples: (**a**) MIL-101-fluorine, (**b**) MIL-101 without treatment, and (**c**) MIL-101 treated with acetic acid; vacuum-heated samples (at 150 °C): (**d**) MIL-101 treated with fluorine, (**e**) MIL-101 without treatment, and (**f**) MIL-101 treated with acetic acid; (**g**–**i**) iDPC-STEM images of the same samples, as shown in (**d**–**f**). In addition, the processed image by real-space averaging (**i**), the simulated projected potential map (**ii**), and the projected structural model (**iii**) are presented for comparison. Reprinted from [112].

When planning the synthesis of MOFs, all parameters must be considered. Li et al. conducted a study of solvothermal synthesis of MOF MIL-101 using hydrofluoric acid and acetic acid as additives. They exposed MIL-101 MOF to vacuum heating after synthesis and used high-resolution transmission electron microscopy (HRTEM) to analyze the results. The study showed that the nature of the acid that was added to the reaction mixture played a crucial role in determining the structure of the size and surface of the MOF (Figure 7) [112]. The HRTEM images revealed that MOFs treated with hydrofluoric acid and untreated MOFs had homogeneous surfaces that were not completely enclosed by middle cells. However, in the case of MIL-101 treated with acetic acid, the surface was entirely composed of cells. In addition, the study proved that vacuum heat treatment could selectively open the surface cells without changing the bulk structure. The optimal temperature for this process depends on the type of additive used during synthesis. This work shows the high possibility of variations in MOF modification for controlled MOF synthesis. Control of morphology and parameters (including surface parameters) plays a key role in the biomedical applications of MOFs [112].

**Figure 8:**
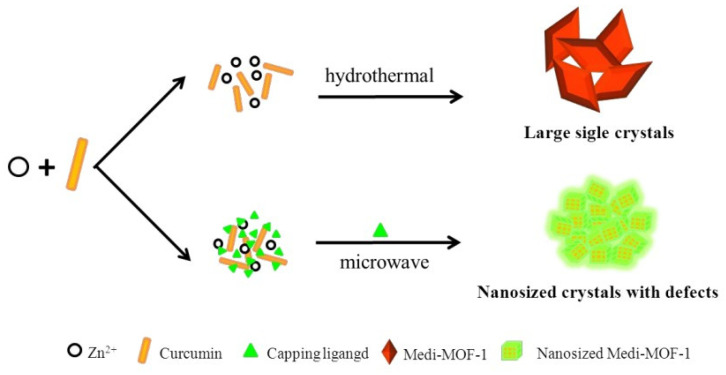
Schematic representation of the synthesis of Medi-MOF-1 by conventional heating and microwave-assisted modulation, reprinted from [113].

Feng et al. demonstrated the possibility of modifying and tuning the morphology of Medi-MOF-1 obtained through microwave synthesis using the modulators 2-Methoxy-4-methylphenol, 4-ethylguaiacol, and isoeugenol while controlling heating time and temperature (Figure 8). When the capping agent was added to the system, there was competitive coordination between curcumin and the blocking agent for zinc ions. Because the capping agent had only one coordination functional group, it blocked the extensive growth of MOF structures. It is worth noting that the capturing agent created defects and mesopores within the crystals, due to the competitive coordination [113]. This work demonstrated the ability to control the pore size and surface morphology. In addition, hierarchical pores of up to 5 nm were formed due to the capturing agents, which can be used to load biomolecules.

## 3. Biomedical Applications of MOFs

The diversity of methods by which MOFs are synthesized (Section 2.1, Section 2.2, Section 2.3, Section 2.4 and Section 2.5), the post-synthetic modification (Section 2.6), and the possible adjustment of key parameters of MOFs (Section 2.7) provide the possibility to prepare a wide range of MOFs with potential biomedical applications, making them versatile and one of the most promising materials of the future. MOFs have been primarily highlighted for therapeutic purposes in drug delivery due to their porous structure and high drug-loading capacity.

MOFs can be used as biodegradable and physiological pH-sensitive systems for photothermal therapy and radiation therapy in the body. With their large surface area, MOFs can also be used to encapsulate large amounts of drugs, while their high structural and functional flexibility allows them to adjust to the shape, size, and functionality of drug molecules. Regarding their potential imaging applications, MOFs can be modified with chemical groups that can affect the delivery of contrast imaging agents. MOFs can act simultaneously as MRI contrast agents and drug carriers, serving both diagnostic and therapeutic purposes. MOFs can also be used in magnetic resonance (MR) and optical imaging by incorporating paramagnetic metal ions and luminescence-based materials, respectively. In addition, prospects have been presented for the development and application of nanoenzymes, mainly derived from MOFs, as antibacterial compounds and in the treatment of cancer. Various authors have also created systems in which nano MOFs (NMOFs) can be successfully used as antibacterial agents, both as prophylactic or therapeutic agents, thus offering significant advantages in diagnosis, monitoring, and therapy. Therefore, in the following sections, given the highly cited potential biomedical applications of nanomaterials [7,8,43,114], we review the potential applications of MOFs.

### 3.1. MOFs as a Therapeutic Agent

**Figure 9:**
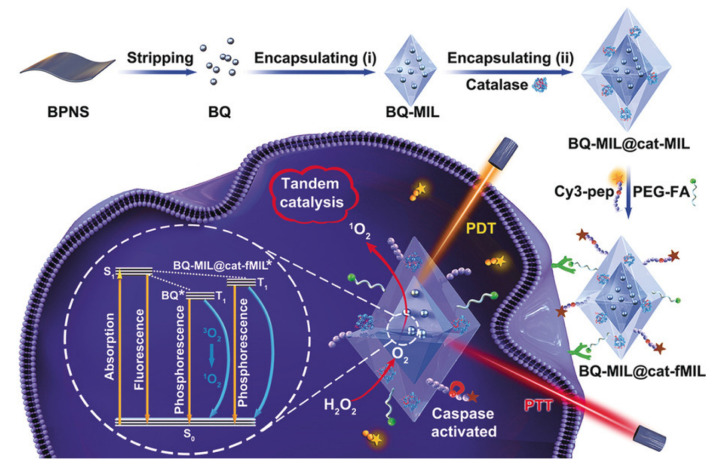
Schematic representation of the preparation of black phosphorus quantum dot (BQ) and catalase in homologous MOFs and its application as a tandem catalyst for enhanced therapy against hypoxic tumor cells, reprinted from [115]. Asterisks are shown after treatment by laser on the MOF heterostructure (BQ-MIL@cat-MIL) in photodynamic therapy.

Liu et al. proposed a hybrid material consisting of MIL-101 modified with black phosphorus quantum dots and catalase. Due to the black phosphorus integrated into the MOF, this system can induce singlet oxygen (^1^O_2_), which can further be used in photothermal and photodynamic therapy of tumor diseases when using IR radiation. The average size of this hybrid system was 140 nm, which allowed it to be used for various organs. This system was delivered to cell lysosomes using the FR-directed effect and the clathrin-dependent endocytosis pathway. It is worth noting that the cell viability of both cancerous HeLa and normal HaCaT cells was over 90%. When irradiated with a laser with a wavelength of 660 and 808 nm (150 mW/cm^2^) for 10 min, the degree of cell apoptosis was 75.6%. The authors noted that the synergistic effect of PDT and PTT markedly induced cell death (Figure 9). They also emphasized the importance of catalase in this system, due to the 8.7-fold increase in the efficiency of PDT, which indicated an increased therapeutic effect against hypoxic tumor cells [115].

**Figure 10:**
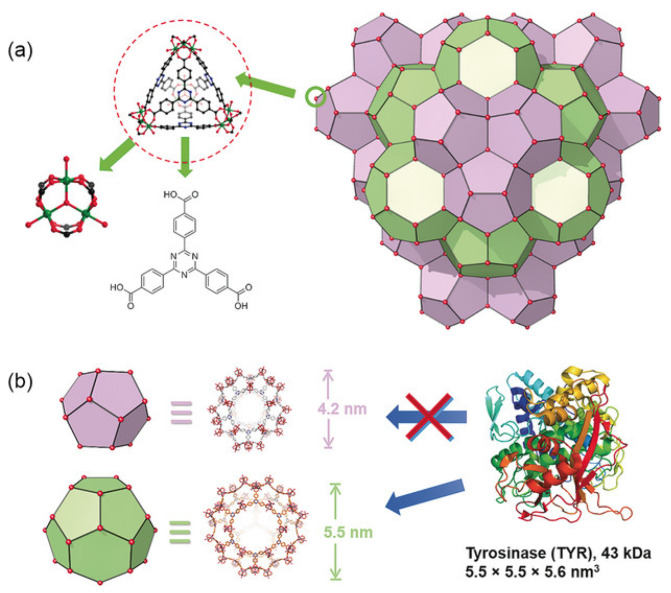
Structure of PCN-333. (**a**) PCN-333 nanoparticles (NPCN-333), (**b**) two types of mesoporous cavities in NPCN-333 of 4.2 nm and 5.5 nm. Given the size of tyrosinase, it can only be loaded in the 5.5 nm pore. Reprinted from [116].

In their work, Lian et al. [116] presented a nanoreactor based on MOF PCN-333 and tyrosinase, as well as paracetamol (added) which was used as a non-toxic prodrug (Figure 10). This system showed high stability, resistance to bodily fluids, and a high loading capacity of 0.8 g/g of MOF. When the nanoreactors based on MOF PCN-333 were exposed to SKOV3-TR cells, it was found that this system was located around the nucleic region (according to confocal microscopy). The synergistic cytotoxic effect of the enzyme included in the MOF, as well as the prodrug in the form of paracetamol concerning SKOV3-TR cells, was proved by in vitro tests. It is worth noting that pure MOF and paracetamol have significantly low cytotoxicity. In addition, in vivo experiments in mice have shown that this system can cause a reduction in tumor size caused by subcutaneous HeLa xenograft as early as the second day. The authors proved that cytotoxicity arises from the enzymatic conversion product of paracetamol and the subsequent formation of reactive oxygen species (ROS) and glutathione depletion (GSH), which cause cancer cell apoptosis/necrosis [116].

**Figure 11:**
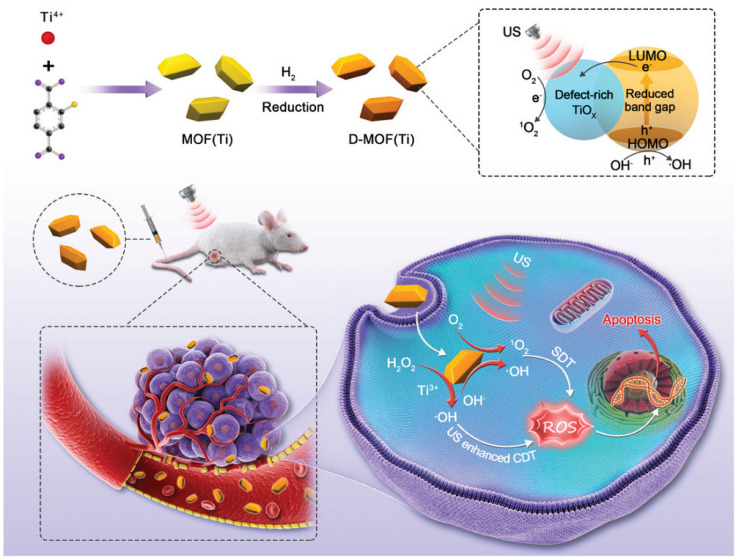
Schematic illustration of synthesis and antitumor therapy of D-MOF(Ti), reprinted from [118].

Sonodynamic therapy is a promising non-invasive cancer treatment that can destroy cancer cells when exposed to sensitizer-induced reactive oxygen species [117]. Liang et al. [118] proposed the use of a defect-rich Ti-based MOF (D-MOF(Ti)) as a sensitizer for sonodynamic therapy. The sonochemical characteristics of D-MOF(Ti) were investigated using a singlet oxygen sensor green (SOSG). After mixing D-MOF(Ti) with the SOSG probe, the intensity of the SOSG fluorescent signal increased significantly with increasing duration of ultrasound irradiation, indicating that ultrasound can stimulate D-MOF(Ti) to produce oxygen. This possibility was also confirmed by EPR spectroscopy. Using the MTT assay, D-MOF(Ti) nanoparticles were found to exert dose-dependent cytotoxicity on 4T1 cells, which may be due to the formation of ROS through the Fenton reaction, causing cell death (Figure 11). Using 2′,7′-dichlorodihydrofluorescein diacetate (DCFH-DA) staining of cells, it was shown that neither MOF nor ultrasound treatment produced sufficient amounts of ROS. In contrast, treatment of the cells with MOF and ultrasound resulted in green fluorescence, indicating a significant improvement in intracellular ROS. In vivo experiments on mice demonstrated that subjects treated with MOF and ultrasound exhibited severe apoptosis and tumor cell necrosis. Due to defects in the nanostructure, D-MOF(Ti) can produce O_2_ during ultrasonic treatment, which can be actively used for the therapy of tumor diseases. Given the low level of hemolysis (less than 2%), relatively low toxicity of MOFs without ultrasound treatment, and a high level of appetite after treatment with ultrasound, the authors suggested that this system is highly promising as a sonosensitizer [118].

### 3.2. Drug Delivery

#### 3.2.1. MOF Carrier

Due to their high porosity, specific surface area, and modifiability, MOFs are a very promising class of compounds for drug delivery. Abanades Lazaro et al. [119] proposed a universal UiO-66 NMOF system based on the simultaneous introduction of carboxylate and phosphonate-containing fragments. This method yields a MOF of approximately 125 nm and enables the incorporation of several drugs simultaneously. The authors demonstrated the possibility of loading alendronate, dichloroacetate, and α-cyano-4-hydroxycinnamic acid into the MOF, and in addition, the MOF was post-synthetically loaded with 5-fluorouracil. They also studied various combinations of drugs included in the MOF. The anti-cancer therapeutic activity of dual drug combinations against MCF-7 breast cancer cells was significantly higher for the three-component systems. A particularly important point is the reduced toxicity of various MOF-based systems to healthy cells compared to free drugs. This will improve safety and cause less harm to the body during antitumor therapy [119]. As a result, they obtained a MOF-based delivery system capable of loading multiple drugs simultaneously. This property allows the loading of drugs for different purposes, which can provide a wide range of therapeutic actions or significantly reduce the burden on the body.

**Figure 12:**
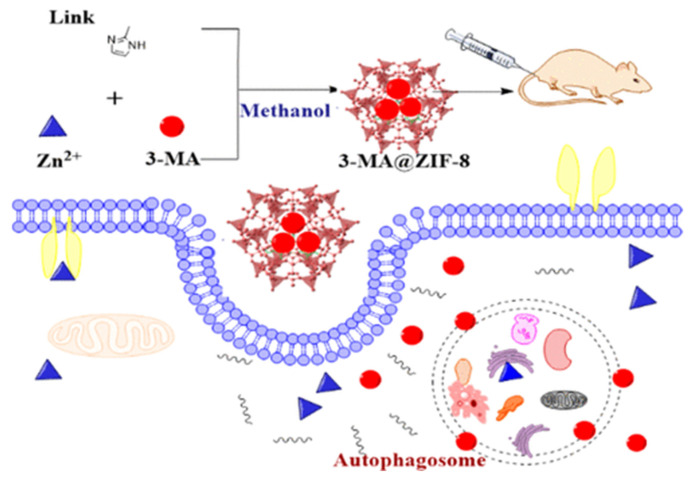
Schematic illustration of the synthesis and delivery of the autophagy inhibitor 3-methyladenine using zinc-imidazole ZIF-8 MOFs, reprinted from [120].

In their work, Chen et al. [120] demonstrated the possibility of controlled delivery of the autophagy inhibitor 3-methyladenine using zinc-imidazole ZIF-8 MOFs (Figure 12). The amount of 3-methyladenine loaded could be controlled by the amount of substance added to the system during synthesis. This system showed pH-dependent drug release. The optimal pH values were approximately 6.5 and 5.0, while at pH 7.4 there was almost no drug release. MOFs loaded with 3-methyladenine showed dose-dependent cytotoxicity in HeLa cells at a particle concentration of 7.5 μg/mL when incubated for 24 h, and mitochondrial function decreased from baseline to 60%. ZIF-8 was localized predominantly in the cytoplasm and subcellular organelles, which was established by transmission microscopy. A study of the biodistribution of MOFs found that Zn accumulation in tissues such as the heart, kidney, and brain was similar to that in the groups treated with physiological solution and 3-methyladenine. However, significantly greater Zn accumulation was observed in the organs of the reticuloendothelial system (RES), including the lungs, liver, and spleen. It is worth noting that the use of ZIF-8 contributes to more accurate control of drug release, more effective antitumor and autophagic inhibition of tumor xenografts, and more moderate toxicity in mice compared to free 3-methyladenine [120].

Non-polar compounds can exhibit anti-nonspecific inflammation, anti-allergic inflammation, anti-tumor effects, and immune regulation, but due to their strong hydrophobic properties, their use is severely limited. For the use of such substances in biomedical practice, especially for oral delivery, special drug delivery carriers are needed, and MOFs can act as such carriers.

Li et al. investigated a strontium-based metal-organic framework as a carrier of the drug ketoprofen to form a complex system for the treatment of osteoarthritis, and the amount of loaded drug and release rate were examined. Ketoprofen was successfully loaded onto the MOF carrier with little or no effect on the crystal structure of the MOF. The amount of ketoprofen Sr-BDC loaded was 36%. Most of the ketoprofen was released after 24 h due to the presence of Sr-BDC on the surface and pores. In addition, the cytotoxicity experiment showed that the synthesized Sr-BDC had no toxic effect on Osteoarthritis chondrocytes [121].

In their research, Zhang et al. proposed Zinc MOF-5 as a carrier for oleanolic acid, which has antitumor activity. The loading rate of MOF-5 with oleanolic acid was 42%. While studying the release kinetics, they found that the active period of drug release was 10 h, after which the rate dropped significantly. After 30 h, the drug release ended, and the maximum drug release was 81.2% in buffer with pH 7.4. In addition, the MOF-oleanolic acid system showed cytotoxicity against ovarian cancer cells at a concentration of 160 µg/mL [122].

#### 3.2.2. Oral Delivery

Li et al. screened the loading capacity of various zirconium MOFs against 5-fluorouracil (5-FU). Zr-MOF and 2,6-naphthalenedicarboxylic acid (Zr-NDC) exhibited the highest loading capacity of 66.28% and 1.3 g/g. The structure of the MOF did not change after loading, as confirmed by the SEM microscopy data. The MOF was subjected to post-synthetic modification with a chitosan solution, which significantly increases the stability of the MOF in biological fluids. The release rates of chitosan-modified MOFs in artificial gastric juice and artificial intestinal fluids were approximately 40% and 75%, respectively, while prolonging the drug release time. In addition, the authors used a pharmacokinetic study to demonstrate that the chitosan-modified MOF system can significantly improve the oral bioavailability of the model drug (5FU) [123].

**Figure 13:**
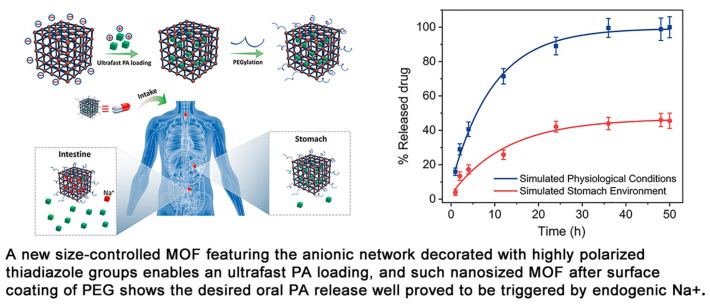
Schematic illustration of the preparation of an anionic MOF, ZJU-64-NSN, for oral drug delivery, reprinted from [124].

Jiang et al. proposed a way to synthesize a new anionic MOF, ZJU-64-NSN, with a controlled size of 300 nm to 200 μm for oral drug delivery. The samples were modified with PEG-NH^2+^ to increase the stability of biological fluids and the affinity of the MOF to the human body (Figure 13). In their work, the authors showed the possibility of controlling MOF size with Zn^2+^ ions and stirring during crystal nucleation. The size control did not affect the crystal structure of the MOF. They demonstrated the possibility of ultrafast loading of procainamide into the MOF and loading of the drug into the MOF at 0.21 g/g for only 1 min (the loading level was maintained regardless of the time of sorption of the drug onto the MOF). ZJU-64-NSN MOF at a concentration of 50 μg/mL was fully nontoxic to rat pheochromocytoma cells (PC12), as proved by the MTT test. Using DAPI staining and confocal microscopy, it was found that at a concentration of 50 μg/mL, an expansion of neurites of living PC12 cells was observed, indicating insignificant cytotoxicity and good biocompatibility of the resulting MOFs [124].

#### 3.2.3. Eye Delivery

The work of Kim et al. demonstrated the ocular delivery of brimonidine using MOF NH2-MIL-88(Fe) obtained by solvothermal synthesis in an autoclave. Brimonidine was loaded into the MOF by stirring for 24 h. The loaded amount of brimonidine was measured as 121.3 µg/mg NH2-MIL-88(Fe). In vitro release experiments in PBS demonstrated a release of approximately 5.2% per h, which was enhanced in the first hours by weakly bound brimonidine molecules. NH2-MIL-88(Fe) and NH2-MIL-88(Fe) with brimonidine incorporated showed no cytotoxic effect, even at ultra-high concentrations (2000 μg/mL). By measuring the zeta potential of MOFs and mucin adsorption, the mucoadhesive properties of the MOFs were confirmed. The authors also studied pharmacokinetic parameters and made a comparison with the drug Alphagan P. In vivo experiments on rabbits showed that after a single injection and after multiple injections of NH2-MIL-88(Fe)/Br into rabbits’ eyes, no obvious complications or damage of the eye tissues were detected, except for mild conjunctivitis [125].

**Figure 14:**
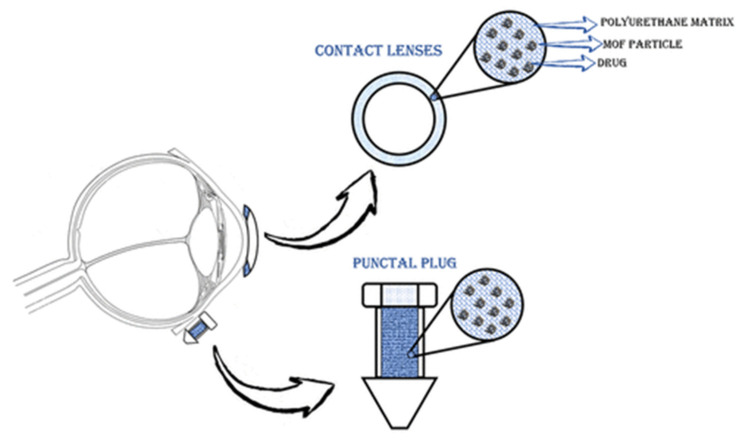
Schematic illustration of the application of polymeric nanocomposite films for drug delivery in ocular therapeutics, either as a component of a contact lens or in punctal plugs. Reprinted from [127].

Gandara-Loe et al. also studied the delivery of brimonidine using different MOFs (HKUST-1, MIL-100 (Fe), UiO-66, and UiO-67). UiO-67 and MIL-100 (Fe) MOFs showed the highest loading of up to 50%. In addition, UiO-67 provided a prolonged release of the drug, as well as low toxicity to retinal cells, which shows the promising potential use of such systems for treating eye diseases [126]. In another work, Gandara-Loe et al. proposed the use of UiO-67 synthesized by the solvothermal method for ocular delivery of brimonidine tartrate (Figure 14). It is worth noting that the drug was delivered using UiO-67 films modified with polyurethane. The loading capacity of the film was 58.4 mg/g after 4 h. Desorption of the drug into the physiological fluids showed a yield of 7% of the loaded drug in the first hour of the experiment, and then approximately 0.3 mg of brimonidine per day, or 4.2 mg for 14 days. It is worth noting that the release of this amount of drug is sufficient for a glaucoma patient. This work confirms that MOF polymer systems can be used for various optical medical devices (contact lenses, tear plugs). These systems have sufficient loading to provide necessary drug delivery to the eye, which conventional films cannot do. This will allow a combination of vision correction and therapy in the future [127].

### 3.3. Diagnostic Systems Based on the MOFs

**Figure 15:**
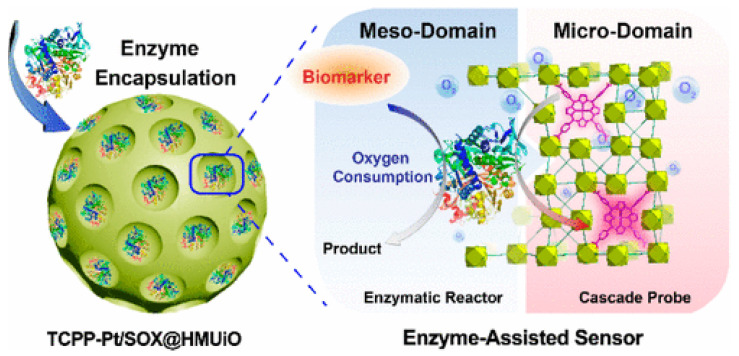
Schematic illustration of a prostate cancer screening system based on UiO-66 MOF, reprinted from [128].

Due to the wide variety of chemical molecules involved in the synthesis of various MOFs, they can be used as diagnostic material for the detection of different molecules/processes. Zhao et al. [128] developed a prostate cancer (PCa) screening system based on UiO-66 MOF (Figure 15). They modified the typical synthesis by adding the surfactant F127 and 1,3,5-trimethylbenzene to regulate the pore size and morphology of the resulting MOF. The O_2_-sensitive luminescent unit TCPP-Pt (phosphorescent carboxylated metalloporphyrin) was attached to the MOF walls via a BDC-NH_2_ linker. In addition, sarcosine oxidase (enzyme) was immobilized in the MOF for a specific enzymatic reaction used for prostate cancer detection. The result was spherical particles with an average size of approximately 100 nm and a distinct UiO-66 crystal structure (BDC-NH2 and TCPP-Pt did not affect the crystal structure of the MOF). The encapsulation of TCPP-Pt in the microporous MOF wall allows it to serve as a cascade signal reporter and prevent the quenching of its aggregation in the aqueous phase. A high level of luminescence was proven in urinalysis of people with PCa, indicating that the probe can effectively respond to sarcosine in urine samples. The authors demonstrated a dose-dependent intensity of luminescence from the concentration of sarcosine in solution, indicating that sarcosine can be quantified to better predict disease in patients [128]. The enzyme and TCPP-Pt molecules are firmly immobilized in the porous substrate, which can effectively prevent their leakage during storage and use. The high accuracy and possibility of determining sarcosine concentrations in biological fluids by a simple luminescence signal make the TCPP-Pt-UiO-66 system highly promising for the detection of PCa.

**Figure 16:**
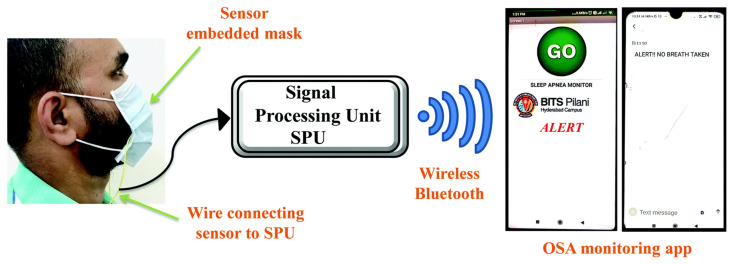
Schematic illustration of the face mask with the embedded HKUST-1–MoS2 device, reprinted from [129].

T. Leelasree et al. proposed using a hybrid system of HKUST-1 MOF and MoS_2_ to create a sensor for detecting sleep apnea problems. A cellulose paper coated with MoS_2_ was ground in a mortar with HKUST-1 MOF and then coated with the resulting mixture. The presence of MOF on the pulp surface was confirmed by X-ray diffraction analysis. This system was used to analyze the subject’s inspiratory rate per minute, which is a key parameter for apnea. In this system, the MOF acts as a universal sorbent for moisture that is released during breathing. This helps to noticeably reduce noise and to detect the respiratory rate with greater clarity. A hybrid sensor based on HKUST-1-MoS2 was prepared on a cellulose material, and the possibilities of creating masks with respiratory rate detection were demonstrated. The system was fully automated and transmitted the signal to an app on a smartphone (Figure 16) [129].

Wang et al. proposed the use of modified MIL-101(Fe) as a contrast agent in MRI diagnostics. After synthesis, the MOF was coated with polylactic acid and PEG. In addition, it could include various drugs that could be used for tumor therapies. An aqueous solution of the modified MOFs showed concentration-dependent MRI darkness and brightness effects, as well as relatively high transverse relaxation and low longitudinal relaxation. The intensity of the fMRI signal was significantly stronger between 9 h and 24 h, suggesting accumulation of MIL-101(Fe) in the tumors because of the increased permeability and EPR retention. Given the possibility of incorporating various drugs into the MOF structure, as well as a low toxicity, this platform has unique prospects as a theranostic object for tumor diseases [130].

**Figure 17:**
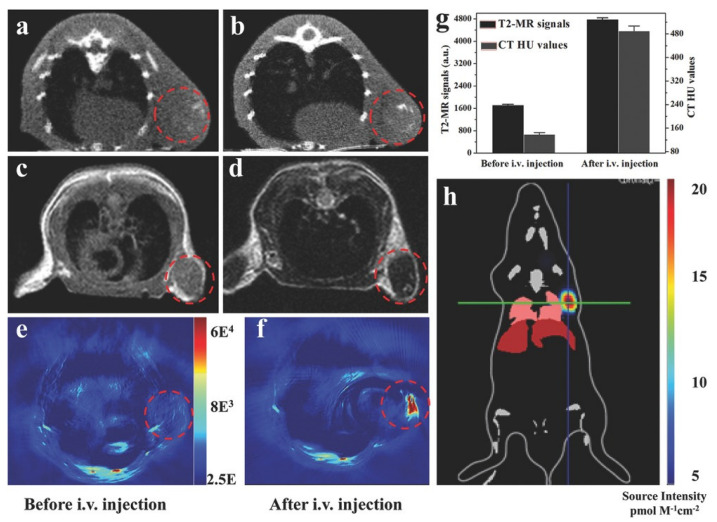
Imaging of U87 MG-subcutaneous tumor-bearing mice. (**a**,**b**) CT images of mice before and 12 h after injection with Au@MIL-88(Fe); (**c**,**d**) MR images of mice before and after injection with Au@MIL-88(Fe); (**e**,**f**) PA imaging of tumors in mice before and 12 h after injection with Au@MIL-88(Fe); (**g**) quantified MRI and CT signals of tumors from mice before and 12 h after injection with Au@MIL-88(Fe); (**h**) bioluminescent imaging of tumor. Reprinted from [131].

In another study, Shang et al. proposed a core-shell structure nanomaterial based on gold nanoparticles and a MIL-88(A) MOF that could be used for glioma diagnosis. The resulting nanocomposites had an average size of approximately 90 nm and a distinct star shape. They demonstrated the possibility of using the MOF–nanoparticle system for multimodal diagnosis of tumor diseases. The CT signals of Au@MIL-88(Fe) solutions depended on the nanoparticle concentration. The intensity of CT signals was 2.4 times stronger 8 h after injection. MRI is a universal method for diagnosing various diseases in various tissues. Contrast agents enhance the signal and allow for the correct diagnosis of tumor localization. The intensity of the tumor MRI signal increased threefold after the MOF injection compared to the baseline. PAI is one of the newer techniques used to visualize cross-sectional views of whole tumors at depths inaccessible to microscopy and with sensitivity levels that cannot be achieved with CT. Contrast at PAI has also been significantly increased with the MIL-88(A)-AuNP system. In addition, this hybrid material showed no cell toxicity. Therefore, this system represents a unique approach for diagnosis using different methods (MRI, CT, PAI) (Figure 17), which enables a comprehensive study of tumor localization [131].

MOF-Gold nanoparticle systems are versatile systems that can be used for the diagnosis of various diseases. Yin et al. demonstrated the detection of β-amyloid oligomers for early diagnosis of Alzheimer’s disease using an aptamer biosensor based on an AuNPs/Fe-MIL-88NH_2_ MOF [132]. Another diagnostic system based on an MOF and gold nanoparticles was an electrochemical immunosensor, in which gold nanoparticles were applied to the electrode surface and coated with MOF-235 with a specific antibody on top. This sensor showed a wide detection range of prostate-specific antigens, which are very important to detect prostate cancer in men [133].

Electrochemical biosensors for in vivo applications are promising materials for the diagnosis of various diseases, including cancer at its earliest stages. El-Sheikh et al. proposed the use of a bimetallic Zn/Ag MOF to diagnose unamplified hepatitis C virus nucleic acid. The Ag/Zn MOF was synthesized using a special linker obtained by adding zinc and silver salts into the solution.

The electrosensor was made by modifying the MOF Ag/Zn HCV probe and bovine serum albumin. The resulting MOF had an average size of 48 to 86 nm (as determined by SEM), which is optimal for in vivo use. The optimal concentration of Ag/Zn MOF was 2 mg/mL, which was determined by studying the effect of different MOF systems on the current strength. The hybridization occurring on the MOF surface was confirmed by optical density measurements at 260 nm before and after hybridization. In addition, incubation experiments were carried out on culturing with random RNA, in which no current shift of the catalytic peak was observed. However, when complementary RNA was added, the signal was markedly enhanced. In addition, Ag/Zn MOF tests were performed on real patient samples, with recovery rates ranging from 90.3% to 100.34% and relative standard deviation ranging from 2.7% to 4.4%,which suggests that this sensor can be applied to a variety of biological fluids [134]. The biosensor based on the bimetallic Ag/Zn MOF has exceptional efficiency, high sensitivity, and a good detection limit, as well as easy practical use.

### 3.4. Other Biomedical Applications of MOFs

**Figure 18:**
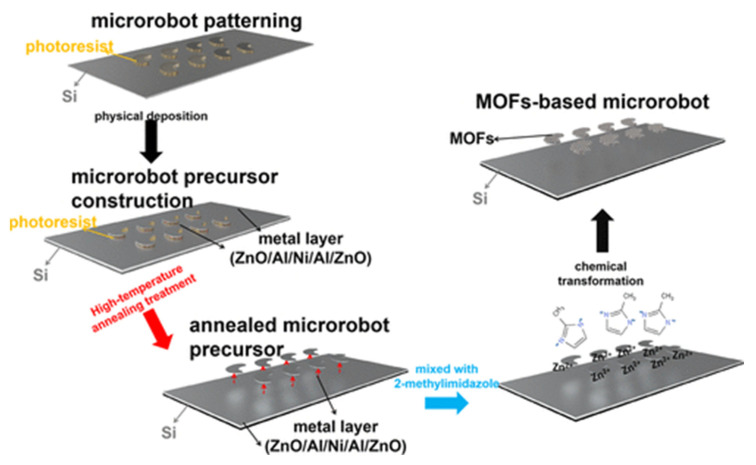
Schematic illustration of the preparation of MOF-based microrobots on Si substrates, reprinted from [135].

In their work, Mu et al. [135] proposed a system for developing microrobots based on a ZIF-8 MOF. The microrobots could have broad applications in various biomedical systems, such as minimally invasive surgery and theranostic systems. ZnO/Al/Ni/Al/ZnO films were successively deposited as microrobot precursors by high-frequency confocal magnetron sputtering and electron-beam vacuum evaporation techniques, after which the precursors were separated from the substrate by calcination. The microrobot precursors were then mixed with a 2-methylimidazole solution to turn them into 2D MOF-based microrobots (Figure 18). The resulting microrobots had a specific surface area of approximately 400 m^2^/g. The authors demonstrated that this system could be controlled using a magnetic field. The ZIF-8-based microrobot showed a high loading capacity for the model drug doxorubicin and demonstrated strong motor abilities, which suggests that this system has great potential for biomedical applications in various areas of the human body [135].

This system shows the transition from MOF to more complex, multifunctional applications that allow not only drug delivery and drug release, but also the creation of metal-organic robots that can solve complex problems.

**Figure 19:**
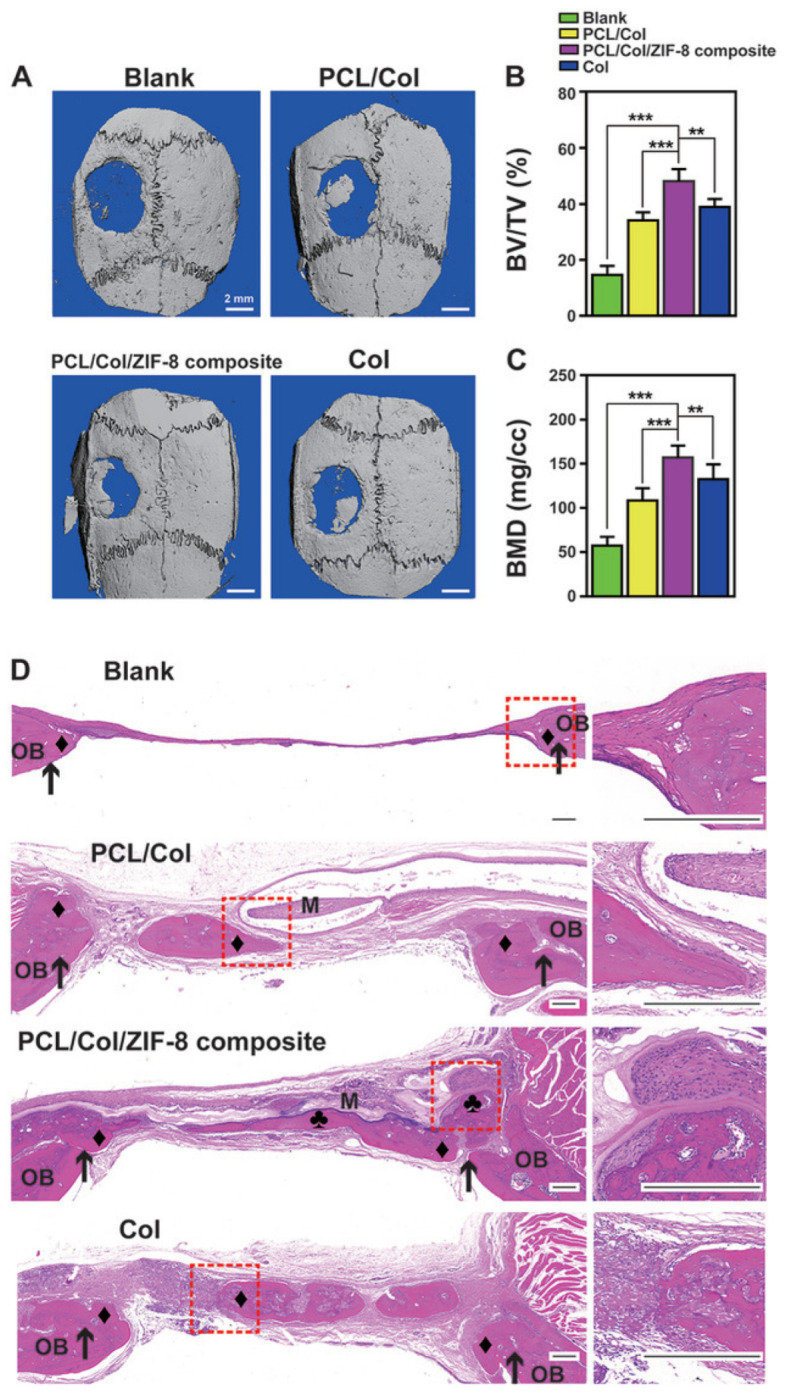
Images of bone formation in a rat cranial defect model after implantation of the PCL/Col/ZIF-8 composite membrane. (**A**) 3D micro-CT reconstructions of the defects 8 weeks post-surgery in different groups; (**B**) bone volume to tissue volume; (**C**) bone mineral density in the calvarial defect determined by micro-CT. Data are presented as mean ± SD (n = 5, ** *p* < 0.01, *** *p* < 0.001); (**D**) H&E staining images of sections of different groups after 8 weeks post-surgery. Around a membrane (“M”), newly formed bone area marked either “♦” (bone grown from defect margin, osteoconductively-generated bones) or “♣” (bone formation initiating from membrane surface, osteoinductively-generated bones). Defect margins indicated by black arrows; OB, old bone. Reprinted from [136].

Xue et al. presented a method for creating a membrane composed of polycaprolactone/collagen (PCL/Col) and ZIF-8 MOF, which can be used for bone tissue engineering. The membrane was fabricated in two stages and modified with ZIF-8 MOF using a hydrothermal process. The resulting systems had an average size of approximately 300 nm according to PEM data. This system provided a continuous release of Zn^2+^ ions in a neutral medium for a week, and in an acidic medium for 12 h. The high biocompatibility was confirmed by the fact that after co-culture with the membrane for 24 h, L929 fibroblasts had a well-stretched morphology with filopodia on the barrier layer and a high viability of 120% of the control. In addition, the ability of the hybrid material to stimulate osteogenesis was proved by quantitative analysis of ALP and ARS activity. The ability of the composite membrane to implant into bone tissue was also evaluated. The membrane consisting of polymers and ZIF-8 adhered to the bone tissue surrounding the defect without additional grafting materials and fixation pins (Figure 19). The ratio of bone volume to tissue volume in the composite group with ZIF-8 MOFs (48.03% ± 4.48%) was markedly higher than in the other and control groups (Figure 19B,C). In addition, the newly formed bone under the polycaprolactone/collagen and ZIF-8 MOF composite membrane was markedly homogeneous and exhibited the highest bone mineral density. In vivo experiments on mice showed no hematological or organ toxicity, indicating a high level of membrane biocompatibility [136].

Liu et al. in their work used bio-MOF-1 to coat a magnesium alloy for bone reconstruction. This coating provided high anti-corrosive properties and increased the biocompatibility of the alloy. It is also worth noting that bio-MOF-1 has biological activity that promotes bone remodeling [137].

In another study, the ability of Mg-MOF modified with a thin layer of CaP and IL4 to intensify osteogenesis and osseointegration was demonstrated. The system had a regenerative effect by optimizing the complex and interconnected microenvironment for healing. It is worth noting that this system was highly biocompatible, and could slowly degrade in biological environments during therapy [138].

In their work, Xu et al. proposed a hybrid system based on CuBDC MOFs for bioactive ion release systems and the therapeutic action of exosomes on bones. In the study, CuBDC was embedded in fibers (lactic acid and glycolic acid) (PLGA), and bone mesenchymal stem cells (hBMSCs-Exo) were fixed on the surface.

The exosomes were physically embedded in the framework and electrostatically interacted with the hybrid MOF fibers, resulting in roughened surfaces with nanoscale particles. The PLGA/CuBDC@Exo hybrid system demonstrated high biocompatibility with CCK-8 cells. It is worth noting that the same cells also had the highest proliferation rate. The expression of proteins related to osteogenic factors (ALP, Runx2, Ocn) and vascular endothelial growth factor (VEGF) clearly increased, which may have been due to the synergistic effect of CuBDC and hBMSCs-Exo. The ALP and ARS staining results also showed that the MOF-modified framework coated with exosomes had excellent osteogenic properties. In addition, it was shown that PLGA/CuBDC@Exo can promote osteogenesis of bones that were fused, and thus improve the formation of new bone that was coated with bone collagen and mature osteocytes [139].

These studies demonstrate that the design of MOFs that mimic the pattern of bone formation during development can provide a strategy to realize a successful regenerative outcome for bone repair. MOF-polymer systems and various biologically active substances can have a synergistic effect that can be further exploited in bone engineering.

## 4. Conclusions

This review presents the importance of conducting comprehensive research on MOFs with a biomedical focus. We systematized the approaches to the synthesis and tuning of optimal parameters (size, specific surface area, porosity, chemical groups), and presented the main methods of obtaining MOFs, which can be used to create diverse biomedical systems based on MOFs. These methods allow for the possibility of tuning key parameters such as surface morphology, which have a significant influence on the scope of specific MOFs. The combination of these measures during the preparation of MOFs with a set of properties facilitates the development of ideal MOFs for biomedical applications. The post-synthetic modification, which can hydrophilize the sample and increase its biocompatibility, can also be considered as another option for creating MOFs for biomedical systems.

In addition to discussing the synthesis and modification of MOFs, we also presented possible biomedical applications of MOFs, including promising methods for pH-sensitive oral and ocular drug delivery. Moreover, MOFs show high potential as carriers for various drugs, both injectable and for oral and ocular drug delivery. We also highlight the possibilities of creating MOF-based hybrid materials for PTT, PDT, and SDT. The wide range of metal ions and ligands, high porosity, and small size make MOFs a promising material for use in diagnostics. In this work, both in vivo and in vitro diagnostic systems based on MOFs were considered. The other promising applications of MOFs include treating skin cancer and bone tissue therapy.

Despite the potential wide range of possible applications of various metal-organic frameworks in biomedicine, we note that MOFs are currently not used in medical practice despite all their distinctive characteristics, according to the database of clinical trials (ClinicalTrials.gov). Therefore, the present review highlights their potential use and the need for future research to test these possibilities. We expect to see an increase in clinical trials on MOFs in the future.

## Data Availability

Data sharing is not applicable.

## Nomenclature

5-FU	5-fluorouracil
ALP	Alkaline phosphatase
ARS	alizarin red S
BDC	benzoldicarboxylate
CT	Computerized tomography
DA	dodecanoic acid
DAPI	4′,6-diamidino-2-phenylindole, a fluorescent stain that binds strongly to adenine–thymine-rich regions in DNA
DCFH-DA	2′,7′-dichlorodihydrofluorescein diacetate
EPR spectroscopy	Electron paramagnetic resonance spectroscopy
GSH	glutathione depletion
HRTEM	high-resolution transmission electron microscopy
IL	ionic liquid
MOF	Metal-organic framework
MRI (fMRI)	Magnetic resonance imaging (functional MRI)
MTT	is a colorimetric assay for assessing cell metabolic activity
NMOF	nano MOFs
PAI	Photoacoustic imaging
Pca	Prostate cancer
PDT	Photodynamic therapy
PEG	polyethylene glycol
PLGA	Copolymer, poly(lactic-co-glycolic acid)
PSM	Post-synthetic modification
PTT	Photothermal therapy
RES	Reticuloendothelial system
ROS	reactive oxygen species
SDT	Sonodynamic therapy
SEM	scanning electron microscopy
SOSG	singlet oxygen sensor green
VEGF	vascular endothelial growth factor
X-ray	nondestructive technique that provides detailed information about the crystallographic structure
